# Neutrophil Extracellular Traps Induce MCP-1 at the Culprit Site in ST-Segment Elevation Myocardial Infarction

**DOI:** 10.3389/fcell.2020.564169

**Published:** 2020-11-09

**Authors:** Thomas M. Hofbauer, Anna S. Ondracek, Andreas Mangold, Thomas Scherz, Johanna Nechvile, Veronika Seidl, Christine Brostjan, Irene M. Lang

**Affiliations:** ^1^Department of Internal Medicine II, Division of Cardiology, Medical University of Vienna, Vienna, Austria; ^2^Department of Dermatology and Venereology, Landesklinikum Wiener Neustadt, Wiener Neustadt, Austria; ^3^Department of Surgery, Division of Vascular Surgery and Surgical Research Laboratories, Medical University of Vienna, Vienna, Austria

**Keywords:** neutrophil extracellular traps, monocyte chemoattractant protein-1, fibrocytes, CCR2, ST-segment elevation myocardial infarction

## Abstract

**Background:**

Leukocyte-mediated inflammation is crucial in ST-segment elevation myocardial infarction (STEMI). We recently observed that neutrophil extracellular traps (NETs) are increased at the culprit site, promoting activation and differentiation of fibrocytes, cells with mesenchymal and leukocytic properties. Fibrocyte migration is mediated by monocyte chemoattractant protein (MCP)-1 and C-C chemokine receptor type 2 (CCR2). We investigated the interplay between NETs, fibrocyte function, and MCP-1 in STEMI.

**Methods:**

Culprit site and peripheral blood samples of STEMI patients were drawn during primary percutaneous coronary intervention. MCP-1 and the NET marker citrullinated histone H3 (citH3) were measured by ELISA while double-stranded DNA was stained with a fluorescent dye. The influence of MCP-1 on NET formation *in vitro* was assessed using isolated healthy donor neutrophils. Human coronary artery endothelial cells (hCAECs) were stimulated with isolated NETs, and MCP-1 gene expression was measured by ELISA and qPCR. CCR2 expression of culprit site and peripheral blood fibrocytes was characterized by flow cytometry. Healthy donor fibrocyte receptor expression and chemotaxis were investigated in response to stimulation with MCP-1 and NETs *in vitro.*

**Results:**

NETs and concentrations of MCP-1 were increased at the culprit site of 50 consecutive STEMI patients. NET stimulation of hCAECs induced transcription of ICAM-1, IL-6, and MCP-1, and secretion of MCP-1. MCP-1 promoted NET formation of healthy donor neutrophils *in vitro*. An increasing MCP-1 gradient correlated with fibrocyte accumulation at the culprit site. Locally increased MCP-1 levels were negatively correlated with CCR2 expression on fibrocytes. MCP-1 and NETs induced CCR2 downregulation on fibrocytes *in vitro*. NETs did not function as a chemotactic stimulus for fibrocytes or monocytes and could block migration in response to MCP-1 for both cell populations.

**Conclusion:**

NETs function as signaling scaffolds at the culprit site of STEMI. NETs assist MCP-1 and ICAM-1 release from culprit site coronary artery endothelial cells. MCP-1 facilitates further NETosis. Monocytes enter the culprit site along an MCP-1 gradient, to transdifferentiate into fibrocytes in the presence of NETs.

## Introduction

ST-segment elevation myocardial infarction (STEMI) accounts for substantial health burden (Hartley et al., 2016; Ibanez et al., 2018) and is a consequence of thrombotic occlusion of coronary arteries (Libby, 2013). The mainstay of treatment today is primary percutaneous coronary intervention to open the coronary vessel and re-establish blood flow to the ischemic myocardium (Ibanez et al., 2018). The target for intervention is fresh thrombus on the surface of atherosclerotic plaques and erosions (Libby et al., 2011). Pathomechanisms of vascular occlusion remain incompletely elucidated. Several immune cell types and subpopulations contribute to coronary thrombosis and amplify ischemia/reperfusion injury and left ventricular remodeling (Maier et al., 2005; Libby et al., 2011; Libby, 2013).

Previously, we have shown accumulation of neutrophils at the culprit site, which was associated with local complement activation (Distelmaier et al., 2009) and could predict mortality (Distelmaier et al., 2014). Neutrophils have the ability to form so-called neutrophil extracellular traps (NETs) in a process different from apoptosis or necrosis, termed NETosis. Several stimulants of NETosis have been described such as Ca^2+^ ionophores, phorbol myristate acetate, IL-8, activated endothelium (Remijsen et al., 2011), oxidized epitopes (Awasthi et al., 2016), and bacteria (Brinkmann et al., 2004). While precise mechanisms are not yet fully understood, however, NET formation seems to be dependent on the presence of reactive oxygen species (ROS) (Fuchs et al., 2007), myeloperoxidase and neutrophil elastase (Metzler et al., 2014), and the calcium-dependent enzyme peptidyl arginine deiminase 4 (PAD-4) (Martinod et al., 2013). Decondensation of nuclear DNA is driven by PAD-4 citrullinating histones (Wang et al., 2004) in synergism with histone degradation by myeloperoxidase and neutrophil elastase (Metzler et al., 2014). The breakdown of intracellular membranes results in adsorption of granular proteins to chromatin before expulsion (Brinkmann et al., 2004). This meshwork of DNA and associated proteins was identified as a major constituent of coronary thrombi (de Boer et al., 2013; Mangold et al., 2015). NET burden was associated with microvascular obstruction, myocardial salvage index, and left ventricular ejection fraction in STEMI patients (Helseth et al., 2019).

Recently, we have reported an impact of NETs on monocytes and fibrocytes at the culprit site, possibly influencing outcome (Hofbauer et al., 2019; Mangold et al., 2019). Fibrocytes are bone marrow-derived cells with properties of mesenchymal cells and leukocytes (Bucala et al., 1994) that can differentiate from monocytes (Keeley et al., 2012). It was proposed that fibrocytes are involved in the pathogenesis of hypertensive (Keeley et al., 2011) and coronary (Medbury et al., 2008) heart disease, including myocardial infarction (Fang et al., 2012). We demonstrated that fibrocytes accumulate and are highly activated at the culprit site in STEMI (Hofbauer et al., 2019), which might be mediated by increased expression of cell adhesion markers and presence of chemoattractants such as monocyte chemoattractant protein (MCP)-1. MCP-1 is recognized by C-C chemokine receptor type 2 (CCR2) expressed on fibrocytes and monocytes and stimulates fibrocyte proliferation, migration, and collagen deposition (Ekert et al., 2011). Elevated MCP-1 was shown to predict all-cause mortality in patients after myocardial infarction (de Lemos et al., 2007). A mutation in the *mcp-1* gene resulting in elevated protein levels in response to inflammatory stimuli (Rovin et al., 1999) was associated with increased susceptibility to ischemic heart disease in a meta-analysis of 22 studies (Cai et al., 2015). Ischemia-triggered release of MCP-1 was reported as a key mechanism of left ventricular remodeling (Frangogiannis et al., 2007). MCP-1 deficiency reduced recruitment of monocytes and fibroblasts and attenuated remodeling, but prolonged the inflammatory phase (Dewald et al., 2005).

In this study, we investigated the role of NETs at the culprit site of STEMI.

## Materials and Methods

Detailed information on used reagents is provided in Supplementary Table S3.

### Sample Collection From Patients and Healthy Controls

We recruited 50 patients with STEMI undergoing primary percutaneous intervention as previously described (Hofbauer et al., 2019). Blood from the culprit site and the arterial sheath was collected. Twenty-one healthy probands served as controls. Blood was immediately processed for flow cytometry and centrifuged at 2000 × *g* for 10 min to receive platelet-poor plasma. Plasma was frozen in aliquots at −80°C for subsequent analysis. This study was approved by the Ethics Committee of the Medical University of Vienna, Austria (approval reference numbers 303/2005, 581/2006, 151/2008, and 114/2011). All participants gave informed written consent. All procedures were performed according to ethical standards of the Declaration of Helsinki 2013.

### Enzymatic Infarct Size

Creatinine phosphokinase isoform MB (CK-MB) area under the curve (AUC) as a measure of enzymatic infarct size was calculated using the trapezoidal formula as previously described (Crimi et al., 2013). CK-MB AUC is indicated as arbitrary units.

### Detection of MCP-1 in Plasma and Cell Culture Supernatants

MCP-1 concentration was measured using the human MCP-1 DuoSet enzyme-linked immunosorbent assay (ELISA, R&D Systems) according to the manufacturer’s instructions. All samples were measured in duplicate. The lower detection limit was 15.6 pg/ml.

### Measurement of NET Surrogate Markers

dsDNA was measured using Sytox^®^ Green Nucleic Acid Stain (1 μM, Invitrogen) in plasma diluted 1:20 in phosphate-buffered saline (PBS) containing 5 mM ethylenediaminetetraacetic acid (EDTA) and 0.1% bovine serum albumin (BSA). Citrullinated histone H3 (citH3) was measured using an ELISA as previously described (Thalin et al., 2017; Hofbauer et al., 2019) by assaying undiluted samples in duplicates.

### Flow Cytometry-Based Quantification of citH3-Positive Neutrophils

Blood collected into K3EDTA-coated tubes was mixed 3:10:5 with anticoagulant buffer (15 nM EDTA, 1% BSA in PBS) and 6% Hetastarch (B| Braun), respectively, and incubated for 40 min at 37°C to allow sedimentation of erythrocytes. The supernatant containing leukocytes was transferred to a new tube filled with PBS and centrifuged at 524 × *g* for 5 min. The pellet was resuspended in 2 ml of RPMI 1640 (Gibco) per 150 μl of blood used in the beginning. Two milliliters was used for stimulation in polystyrene round-bottom tubes. Cells were treated with recombinant human MCP-1 (BioLegend, 500 pg/ml) or left untreated for 2:30 h at 37°C. Tubes were gently vortexed before fixation with 1% formaldehyde. Two thirds of the suspension was transferred to a new tube and mixed with 500 μl of FC buffer (2% BSA in PBS) before centrifugation at 3200 × *g* for 15 min at 4°C. The pellet was resuspended in red blood cell lysis buffer (154 mM ammonium chloride, 10 mM potassium bicarbonate, and 0.1 mM disodium EDTA, pH 7.3) and incubated for 15 min. After centrifugation, unspecific binding sites were blocked with FC buffer. All antibodies were diluted in FC buffer and washing between incubations was done by centrifugation at 3200 × *g* for 15 min at 4°C. The primary antibody directed against citH3 (Abcam) was added for 20 min at a concentration of 5.5 μg/ml. Thereafter, the secondary antibody (goat anti-rabbit-Alexa Fluor 647, 1:10,000, Invitrogen) and CD66b-PacificBlue (BioLegend, 1:40) were added for 20 min. Cells were resuspended in FC buffer. Acquisition and analysis of 50,000 events was performed using the Attune NxT Flow cytometer (Life Technologies) and accompanying software. Neutrophils were defined as cells positive for CD66b. CitH3-positive neutrophils were gated with respect to unspecific binding of the secondary antibody and are given as percentage of total neutrophils.

### Isolation of Neutrophils

Neutrophils were isolated as previously described (Brinkmann et al., 2010). 6 ml of whole blood was layered carefully on top of 6 ml of Histopaque 1119 (Sigma) and centrifuged at 800 × *g*, 20 min, brakes off. After harvesting the neutrophil layer, cells were washed once with sterile PBS. The neutrophil pellet was resuspended in PBS and layered on top of a Percoll gradient (Sigma). Therefore, 18 ml of Percoll was mixed with 2 ml of PBS (10 ×) and density gradients of 85, 80, 75, 70, and 65% were prepared with PBS (1 ×). The gradients were layered with decreasing density before resuspended cells were added on top and centrifuged as above. Purified neutrophils were harvested and washed with PBS, and resuspended in Hank’s balanced salt solution (HBSS) without phenol red (Lonza) containing 1.26 mM CaCl_2_. The purity of neutrophils was assessed using a XN-350 Hematology Analyzer (Sysmex) and was consistently above 95%.

### DNA Release Assay to Detect NET Formation

NET formation of isolated healthy donor neutrophils was measured using Sytox^®^ Green Nucleic Acid Stain (Invitrogen), which is cell-impermeable and exclusively stains extracellular DNA as previously described (Vong et al., 2013). Isolated neutrophils (1 × 10^5^) were seeded in duplicates into a 96-well flat-bottom culture plate. As a positive control, a final concentration of 0.33% Triton X-100 was added to one duplicate leading to maximum release of DNA. After incubation with recombinant human MCP-1 (BioLegend, 125, 250, and 500 pg/ml) or vehicle control for 20 min, NET formation was induced with 1.3 μM ionomycin (Sigma) for 2:45 h at 37°C, 5% CO_2_. Sytox Green (Invitrogen) was added to a final concentration of 5 μM for 15 min, and fluorescence was measured on a Promega GloMax Discover microplate reader (excitation 485 nm, emission 520 nm) to assess release of dsDNA into the supernatant.

### Intracellular Calcium Mobilization by MCP-1

Intracellular calcium mobilization was monitored using the Fluo-8 No Wash Calcium Assay Kit (Abcam) according to the manufacturer’s instructions for non-adherent cells. Healthy donor neutrophils isolated as described above were resuspended in equal amounts of HBSS supplemented with 1.26 mM CaCl_2_ and 20 mM HEPES and Fluo-8 dye-loading solution. A total of 10^5^ cells/well/100 μl were seeded into sterile, black, flat-bottom 96-well plates and incubated for 30 min. Pre-warmed HBSS supplemented with 1.26 mM CaCl_2_ was added to a final assay volume of 270 μl. The plate was transferred into a fluorescence plate reader with a dual injector function (Varioskan Flash, Thermo Scientific) and monitored at 37°C for 85 min reading fluorescence at an excitation wavelength of 490 nm and an emission wavelength of 525 nm. After baseline recording, MCP-1 was injected at medium speed (BioLegend, 0.5 ng/ml final concentration) and fluorescence was measured per well immediately afterward. The plate was scanned every minute for 20 min. Then, ionomycin was injected at medium speed (Sigma, 1.3 μM final concentration) and fluorescence was again measured per well immediately afterward. Calcium mobilization was monitored for 15 min every minute and then for 50 min every 5 min. Results are presented as fold change to baseline.

### Intracellular Formation of ROS by MCP-1

Intracellular formation of ROS was monitored using the DCFDA Cellular ROS Detection Assay Kit (Abcam) according to the manufacturer’s instructions for non-adherent cells. Healthy donor neutrophils isolated as described above were resuspended in 1 × assay buffer containing 20 μM DCFDA. A total of 10^5^ cells/well/100 μl were seeded into sterile, black, flat-bottom 96-well plates and incubated for 30 min. Pre-warmed HBSS supplemented with 1.26 mM CaCl_2_ was added to a final assay volume of 270 μl. The plate was transferred into a fluorescence plate reader with a dual injector function (Varioskan Flash, Thermo Scientific) and monitored at 37°C for 85 min reading fluorescence at an excitation wavelength of 490 nm and an emission wavelength of 525 nm. After baseline recording, MCP-1 was injected at medium speed (BioLegend, 0.5 ng/ml final concentration) and fluorescence was measured per well immediately afterward. The plate was scanned every minute for 20 min. Then, ionomycin was injected at medium speed (Sigma, 1.3 μM final concentration) and fluorescence was again measured per well immediately afterward. ROS formation was monitored for 15 min every minute and then for 50 min every 5 min. Results are presented as fold change to baseline.

### NETs Generation and Harvest

NETs were isolated as previously described (Hofbauer et al., 2019), with modifications. Briefly, 5 × 10^6^/ml neutrophils isolated from healthy donors as described above were seeded into 6-well flat-bottom cell culture plates in RPMI supplemented with 3% fetal calf serum (FCS) and stimulated with 500 nM phorbol myristate acetate (Sigma) for 4 h at 37°C, 5% CO_2_. After discarding supernatant, generated NETs were incubated with PBS containing 10 U/ml of the restriction enzyme *Alu*I (Roche) for 30 min. The supernatant was collected and centrifuged at 300 × *g* for 5 min to remove cellular debris. Supernatant was again collected, and double-stranded DNA concentration was measured using the Quant-iT PicoGreen kit (Thermo Fisher) as previously described (Hofbauer et al., 2019).

### Stimulation of Human Coronary Artery Endothelial Cells

Human coronary artery endothelial cells (hCAECs, Lonza) were seeded into flat-bottom 12-well plates at a concentration of 4 × 10^4^/ml in M199 medium (Sigma) supplemented with 20% FCS (Merck), 25 mM HEPES (gibco), 2 mM L-glutamine (Lonza), 1% MEM non-essential amino acids (Sigma), 100 U/ml penicillin, 100 U/ml streptomycin, 2.5 ng/ml amphotericin B (Lonza), and endothelial cell growth supplement (bovine thalamus homogenate supplemented with sodium chloride, streptomycin, ammonium phosphate, and sodium phosphate). After reaching confluence, cells were stimulated using 500 ng/ml isolated NETs, 1 IE/ml deoxyribonuclease (DNase) 1 (Pulmozyme^®^, Roche), 250 ng/ml citH3 (Cayman Chemical), and 250 ng/ml lambda DNA (Thermo^TM^ Fisher) for 6 and 24 h. The supernatant was collected, centrifuged for 10 min at 10,000 × *g* to remove cellular debris, and stored at −80°C for subsequent analysis. Cells were lysed using 500 μl of TRIzol reagent (ThermoFisher) and stored at −80°C.

### Isolation of RNA

RNA was isolated from cell lysates using the ReliaPrep^TM^ RNA Cell Miniprep System (Promega) with modifications. Samples were incubated 5:1 in chloroform for 3 min and centrifuged for 15 min, 12,000 × *g* at 4°C. The aqueous, RNA-containing phase was transferred to a minicolumn and centrifuged for 1 min, 14,000 × *g* at 21°C. After washing with RNA washing solution, aqueous phase was incubated with Yellow Core Buffer containing DNase 1 and MnCl_2_ (both 1:10) for 15 min. After three further washing steps, isolated RNA was eluted in nuclease-free water. RNA concentration was assessed using a NanoDrop^®^ ND-1000 Spectrophotometer (Peqlab).

### cDNA Synthesis

cDNA was synthesized from RNA isolates using the GoScript^®^ Reverse Transcription System (Promega) according to the manufacturer’s instructions. The mix contained oligo(dT)15 as well as random primers. Five hundred nanograms of RNA was transcribed into cDNA in a volume of 20 μl, incubating the samples for 5 min at 25°C, 60 min at 42°C, and 15 min at 70°C followed by a cool down to 4°C in an Eppendorf Mastercycler ep Gradient S.

### Real-Time Quantitative PCR Analysis

Reverse transcriptase quantitative polymerase chain reaction (RT-qPCR) analyses were performed using a GoTaq Probe 2-step RT-qPCR System (Promega) according to the manufacturer’s instructions. All measurements were made on an ABI PRISM 7000 Sequence Detector (Applied Biosystems). Efficiency of primer probe pairs was measured by assaying a six-point 1:2 serial dilution of pooled hCAEC cDNAs, using the same instrument settings as for all other experiments. Amplification efficiency (*E*) was calculated using the formula *E* = 2^–1/–^*^*y*^*, where *y* is the slope of the standard curve resulting from plotting Cq values against dilution (Supplementary Figure S1). Suitable endogenous controls were identified by analyzing expression of actin beta (ACTB), glyceraldehyde 3-phosphate dehydrogenase (GAPDH), and hypoxanthine phosphoribosyl transferase 1 (HPRT1) of one exemplary stimulation experiment according to published recommendations (Chervoneva et al., 2010; Kozera and Rapacz, 2013), using the same instrument settings as for all other experiments. ACTB and HPRT1 were identified to be adequate endogenous controls due to similarity of amplification curves with experimental assays and lack of gene regulation by experimental conditions. Representative plots are given in Supplementary Figure S2. Relative expression levels were calculated using the Pfaffl method (Pfaffl, 2001). The following primers (all ThermoFisher) were used: ACTB (Hs99999903), GAPDH (Hs99999905), HPRT1 (Hs99999909), intercellular adhesion molecule-1 (ICAM-1, Hs00164932), interleukin-6 (IL-6, Hs00174131), and MCP-1 (Hs00234140).

### Identification of Circulating Fibrocytes by Flow Cytometry

Fibrocytes were analyzed using flow cytometry as previously described (Hofbauer et al., 2019). EDTA whole blood samples were incubated with fluorochrome-labeled primary antibodies against collagen-I (1:200, Merck-Millipore), CD34 (1:40, BioLegend), CD45 (1:40, BioLegend), and CCR2 (1:40, BioLegend) for 15 min. After red blood cell lysis (BD), cells were washed twice with PBS and analyzed on a BD FACSCanto II and FACSDiva Software (BD). Debris was excluded using forward and side scatter. Fibrocytes were identified as cells triple-positive for the leukocyte marker CD45, the hematopoietic stem cell marker CD34, and collagen-I (Pilling et al., 2009). Expression levels are given as mean fluorescence intensity (MFI).

### Isolation of Peripheral Blood Mononuclear Cells

For isolation of peripheral blood mononuclear cells (PBMCs), 20 ml of healthy control whole blood was mixed with 10 ml of PBS, layered onto 15 ml of Lymphocyte Separation Medium (PromoCell), and centrifuged for 30 min, 800 × *g* at 21°C, with brakes off. The PBMC layer was harvested and washed with PBS. The remaining red blood cells were lysed using 154 mM ammonium chloride, 10 mM potassium hydrogen carbonate, and 0.1 mM EDTA (pH 7.3); washed twice; and resuspended in RPMI-1640 medium (Sigma). PBMC purity was assessed using an XN-350 Hematology Analyzer (Sysmex).

### *In vitro* Stimulation of Fibrocytes

Isolated PBMCs were resuspended at a concentration of 1 × 10^6^ cells/ml in RPMI-1640 (Sigma), seeded into polystyrene tubes, and stimulated with recombinant MCP-1 (BioLegend, 0.125, 0.250, 0.500, and 5 ng/ml), isolated NETs of four healthy donors (500 ng/ml dsDNA content), and corresponding control supernatant of unstimulated neutrophils for 6 h at 37°C, 5% CO_2_. Afterward, cells were washed twice with PBS and unspecific binding sites were blocked with Fc fragments (BD Biosciences). Samples were incubated with fluorochrome-labeled primary antibodies against collagen-I (1:100, Merck-Millipore), CD34 (1:100, BioLegend), CD45 (1:240, BioLegend), and CCR2 (1:50, BioLegend) for 15 min. Cells were fixed with BD FACS lysis (BD), washed twice with PBS, and analyzed with an Attune NxT flow cytometer (Life Technologies). Debris was excluded using forward and side scatter. Fibrocytes were identified as described above. CCR2 expression levels are given as MFI relative to baseline.

### Cell Migration Experiments

Chemotaxis assays were carried out in 24-well plates with cell culture inserts separating the upper and lower compartment by a membrane of 8-μm pore size (Greiner Bio-One). Chemotactic stimuli were added to the wells in RPMI supplemented with 0.3% BSA (Sigma). FCS (20%) served as assay positive control of migration. Recombinant MCP-1 (BioLegend, 0.500 ng/ml), isolated NETs of four healthy donors (500 ng/ml dsDNA content), and corresponding volume of control supernatant of unstimulated neutrophils were used to stimulate chemotaxis. The PBMC suspension (2 × 10^6^ cells) isolated from 10 corresponding donors was added to inserts with or without NETs and control supernatant. After 6 h at 37°C, the remaining cells were aspirated from the inner side of the insert and discarded, while cells from the lower chamber and the bottom side of the membrane were harvested using Trypsin EDTA for analysis by flow cytometry. Cells were washed twice with PBS and unspecific binding sites were blocked with Fc fragments (BD Biosciences). Samples were incubated with fluorochrome-labeled primary antibodies against collagen-I (1:100, Merck-Millipore), CD34 (1:100, BioLegend), CD45 (1:240, BioLegend), and CD14 (1:50, BioLegend) for 15 min. Cells were washed two times with PBS and analyzed with an Attune NxT flow cytometer (Life Technologies). Debris was excluded using forward and side scatter. Fibrocytes were identified as described above. Cells not characterized as fibrocytes and positive for CD45 and CD14 were identified as monocytes. Cell counts per microliter were recorded.

### Statistics

Normality of data was analyzed via histograms and the Kolmogorov-Smirnov test (data not shown). In case of normal distribution, demographical data are presented as mean ± standard deviation (SD); otherwise, median and interquartile range (IQR) are given. Two groups were compared with respect to matching, normality, and variance, and multiple testing was corrected using the Bonferroni–Holm method. For patient data, all comparisons, uncorrected and corrected *p* values, as well as the specific tests employed are reported in Supplementary Tables S1, S2. Comparison of more groups was done with one-way ANOVA considering normality and repeated measures, if applicable, followed by Tukey’s or Dunn’s multiple comparisons test. Correlations were calculated using Spearman’s rank correlation. Statistical tests used are specified in the respective figure legends. To identify determinants of enzymatic infarct size, we computed a multivariable linear regression model. Normality of residuals was verified using histograms and P–P and Q–Q plots. Heteroscedasticity was excluded by plotting the model residuals versus predicted values. Autocorrelation of residuals was assessed by Durbin–Watson statistics, which was inconclusive (1.812). Multicollinearity between predictors was excluded. Statistical analyses were performed using IBM SPSS 26.0 and GraphPad Prism 8.0 for Windows. Boxplots display the 25th and 75th percentile; whiskers are defined according to Tukey.

## Results

### Culprit Site Milieu as Determinant of Enzymatic Infarct Size

We studied 50 patients presenting with STEMI. Patient characteristics are shown in Table 1.

**TABLE 1:** Patient characteristics.

Patient characteristics	*n* = 50
Age, years ± SD	61 ± 12
Male sex, *n* (%)	39 (78)
BMI > 25 kg/m^2^, *n* (%)	34 (68)
BMI > 30 kg/m^2^, *n* (%)	9 (18)
Diabetes, *n* (%)	9 (18)
History of hypertension, *n* (%)	37 (74)
Dyslipidemia, *n* (%)	33 (66)
Ever smoker, *n* (%)	33 (66)
Family history of CAD, *n* (%)	24 (48)
Previous MI, *n* (%)	9 (18)
**Culprit lesion, *n* (%)**	
LAD	24 (48)
CX	8 (16)
RCA	16 (32)
Multiple	2 (4)
**CAD, *n* (%)**	
1-VD	23 (46)
2-VD	13 (26)
3-VD	14 (28)
Symptom to balloon time, min	194 [146–415]
CK-MB AUC	8533 [4049–15,570]
CRP, nmol/L (< 4.8)	3.62 [1.90–7.81]
TnT, μg/L (0–0.03)	0.05 [0.02–0.11]
Creatinine, μmol/L (50–100)	86.16 ± 29.74
Cholesterol, mmol/L (< 5.2)	4.99 ± 0.85
LDL, mmol/L (< 4.1)	2.74 ± 0.83
HDL, mmol/L (> 1.5)	1.22 ± 0.34
Triglycerides, mmol/L (< 1.7)	1.61 ± 1.12
Culprit site dsDNA, ng/ml	529.8 [428.9–739.8]
Peripheral dsDNA, ng/ml	403.7 [349.2–562.7]
Culprit site citH3, ng/ml	331.6 [122.6–810.5]
Peripheral citH3, ng/ml	235.3 [112.8–434.3]

*Characteristics of patients with STEMI are indicated with respective units and reference values in parentheses thereafter. Data are presented as mean ± standard deviation (SD), median [interquartile range (IQR)], or number (percent) of patients. Culprit site and peripheral NET markers differ significantly (dsDNA, *p* < 0.0001; citH3, *p* = 0.0027; Wilcoxon matched-pairs signed rank test). ASA, acetylsalicylic acid; BMI, body mass index; CAD, coronary artery disease; CK-MB AUC, area under the curve of creatinine kinase isoform MB; CRP, C-reactive protein; CX, circumflex; HDL, high-density lipoprotein; LAD, left anterior descending; LDL, low-density lipoprotein; MI, myocardial infarction; RCA, right coronary artery; STEMI, ST-segment elevation myocardial infarction; TnT, troponin T; VD, vessel disease.*

**FIGURE 1:**
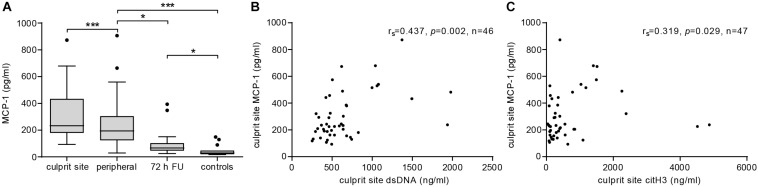
MCP-1 and NETs are elevated at the culprit site in STEMI patients. **(A)** MCP-1 levels were measured in samples from culprit site (*n* = 47), peripheral site (*n* = 47), peripheral blood 72 h follow-up (*n* = 19), and healthy controls (*n* = 15) by ELISA. Statistical tests for respective pairwise comparisons and corrected and uncorrected *p* values are provided in Supplementary Table S1. Data are presented as boxplots with whiskers defined according to Tukey. Culprit site MCP-1 concentrations were positively correlated with **(B)** local dsDNA (Spearman signed-rank test, *r*_*s*_ = 0.437, *n* = 46) and **(C)** citH3 levels (Spearman signed-rank test, *r*_*s*_ = 0.319, *n* = 47). *p < 0.05, **p < 0.01, ***p < 0.001.

Plasma MCP-1 levels were significantly elevated at the culprit site compared to the concentration at the peripheral femoral site (Figure 1A). Concentrations declined over the following 72 h but were still significantly higher than levels measured in samples from healthy controls. To assess the influence of MCP-1 levels on infarct size, a multiple linear regression model with CK-MB AUC as outcome variable was calculated (*n* = 26). The *R*^2^ for the overall model was 0.667 (adjusted 0.538), which illustrates a high goodness of fit (Table 2). The model predicted enzymatic infarct size [*F*(7,18) = 5.157, *p* = 0.002] and culprit MCP-1 levels were significantly added to enzymatic infarct size, with a standardized β coefficient of 0.647. Culprit site MCP-1 correlated with local levels of the NET markers dsDNA (Figure 1B, *r*_*s*_ = 0.437, *p* = 0.002) and citH3 (Figure 1C, *r*_*s*_ = 0.319, *p* = 0.029).

**TABLE 2:** Determinants of enzymatic infarct size.

	β coefficient	[95% CI]	Standardized β	*p*-value
Age	186.2	[1.237; 371.1]	0.317	0.049
Female sex	−1842	[−7458; 3773]	−0.100	0.499
BMI	−847.7	[−1541; −153.7]	−0.358	0.019
Smoker	4796	[−306.4; 9898]	0.278	0.064
Anterior infarction	5272	[−186; 10732]	0.285	0.058
Symptom to balloon time	9.634	[2.685; 16.58]	0.424	0.009
Culprit lesion MCP-1	36.13	[18.74; 53.51]	0.647	<0.001

*MCP-1 levels significantly add to enzymatic infarct size. A multiple linear regression model with CK-MB AUC as outcome variable was calculated (*n* = 26). The *R*^2^ for the overall model was 0.667 (adjusted 0.538), which is indicative of a high goodness of fit. The model significantly predicts enzymatic infarct size *F*(7, 18) = 5.157, *p* = 0.002. CK-MB AUC, creatinine kinase isoform MB area under the curve.*

### NETs Induce a Pro-inflammatory Phenotype in Human Coronary Endothelial Cells

**FIGURE 2:**
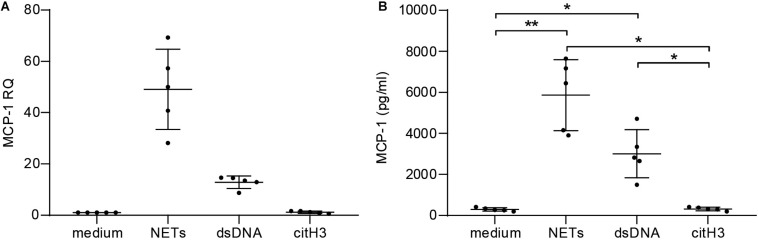
Stimulation of human coronary artery endothelial cells with NETs. hCAECs were stimulated with isolated NETs, dsDNA, and citH3 for 6 and 24 h. MCP-1 gene expression was assessed **(A)** on mRNA levels by qPCR after 6 h and is presented as fold change over an unstimulated control after normalization to the mean of two endogenous controls. **(B)** Release of MCP-1 was measured in cell supernatants by ELISA after 24 h of stimulation. Data are presented as mean ± SD. Experiments were repeated five times and analyzed using one-way ANOVA followed by Tukey’s multiple comparisons test. *p < 0.05, **p < 0.01.

To assess the pro-inflammatory effect of NETs *in vitro*, hCAECs were cultured and stimulated with NETs from healthy donors. Relative quantification by qPCR revealed that NETs (50-fold) and dsDNA (10-fold) induced MCP-1 expression on an mRNA level (Figure 2A) and led to significantly elevated protein levels in cell culture supernatants, as measured by ELISA (Figure 2B). Furthermore, NETs led to a more than 100-fold upregulation of the adhesion marker ICAM-1 in response to NETs (Supplementary Figure S3A), which was accompanied by an 8-fold increase of IL-6 mRNA (Supplementary Figure S3B). Expression of both markers was lower using dsDNA as a stimulant, but still led to a 20-fold increase of ICAM-1 and a 5-fold elevation of IL-6 mRNA, respectively. The addition of DNase did not antagonize the effects of NETs for any of the analyzed targets (data not shown), and citH3 alone did not confer any changes (Figures 2A,B and Supplementary Figures S3A,B).

### MCP-1 Primes Neutrophils for NET Formation

**FIGURE 3:**
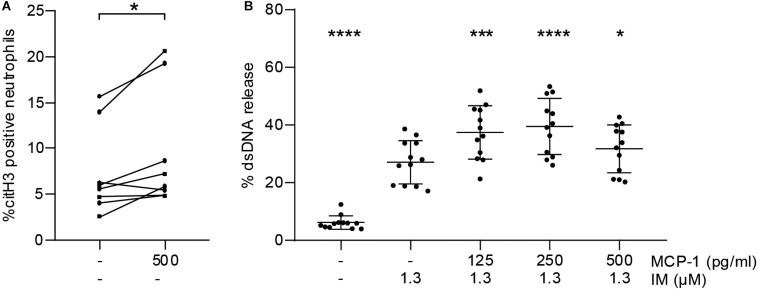
MCP-1 primes neutrophils for NET formation. **(A)** Leukocytes of healthy donors (*n* = 8) were treated with 500 pg/ml of MCP-1 for 2:30 h and analyzed by flow cytometry. Data are presented as percentage of CD66b-positive cells staining for citH3. Groups were compared by Wilcoxon matched-pairs signed rank test. **(B)** Neutrophils of healthy donors (*n* = 12) were pre-treated with 125, 250, and 500 pg/ml MCP-1 and then stimulated with 1.3 μM ionomycin (IM) to induce formation of NETs. Data are provided in percent of positive control and are presented as mean ± SD. Experiments were analyzed by repeated measures ANOVA followed by Dunnett’s multiple comparisons test using the group treated with 1.3 μM IM as single comparator. *p < 0.05, ***p < 0.001, ****p < 0.0001.

To test whether MCP-1 primes neutrophils to undergo NETosis *in vitro*, we measured citH3 by flow cytometry. Stimulation with MCP-1 led to an increase in the percentage of citH3 positive neutrophils compared to untreated controls (Figure 3A). This observation did not directly translate into release of NETs as measured by dsDNA (Supplementary Figure S4A). We then assessed whether pre-treatment of isolated neutrophils with MCP-1 would modulate NET formation in response to a second stimulus. Release of dsDNA *in vitro* using ionomycin was significantly promoted by MCP-1 (Figure 3B). The highest level of NET formation occurred after pre-treatment with 250 pg/ml MCP-1, corresponding to levels measured at the culprit site in STEMI patients. Intracellular mobilization of Ca^2+^ and formation of ROS were not affected by MCP-1 in our setting (Supplementary Figures S4B,C).

### Fibrocyte Influx Is Dependent on MCP-1 Gradient and CCR2 Trafficking

**FIGURE 4:**
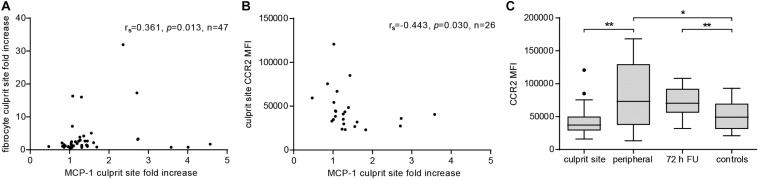
Effect of MCP-1 on accumulation of fibrocytes and fibrocyte CCR2 expression at the culprit site. Fibrocyte counts in culprit and peripheral site blood, presented as influx ratio at the culprit site, and mean fluorescence intensity (MFI) of CCR2 expression were determined by flow cytometry; MCP-1 was measured by ELISA and presented as fold increase at the culprit site. **(A)** Correlation of relative fibrocyte count at the culprit site with MCP-1 fold increase (Spearman signed-rank test, *r*_*s*_ = 0.361, *n* = 47). **(B)** Correlation of fibrocyte CCR2 expression at the culprit site with MCP-1 fold increase (Spearman signed-rank test, *r*_*s*_ = –0.443, *n* = 26). **(C)** CCR2 receptor expression on fibrocytes of culprit site (*n* = 26), peripheral site (*n* = 26), 72 h follow-up (*n* = 14), and healthy control blood (*n* = 18). Statistical tests for respective pairwise comparisons, and corrected and uncorrected *p* values are provided in Supplementary Table S2. Data are presented as boxplots with whiskers defined according to Tukey. *p < 0.05, **p < 0.01.

**FIGURE 5:**
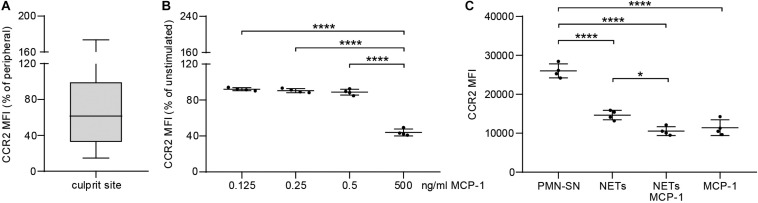
Effect of MCP-1 and NET stimulation on fibrocyte CCR2 expression *in vivo* and *in vitro.* **(A)** Culprit site CCR2 expression on fibrocytes is presented relative to peripheral CCR2 expression. **(B)** PBMCs of healthy donors were stimulated for 6 h with 0.125, 0.250, 0.500, and 5 ng/ml recombinant MCP-1 and analyzed for CCR2 expression by flow cytometry. Data are presented relative to the unstimulated control as mean ± SD. **(C)** PBMCs of healthy donors were stimulated for 6 h with NETs (500 ng/ml dsDNA content), MCP-1 (5 ng/ml), NETs and MCP-1, and neutrophil control supernatant (PMN-SN) to investigate the effect on CCR2 expression as mean fluorescence intensity (MFI). Data are presented as mean ± SD. Experiments were repeated with four different donors and analyzed by repeated measures one-way ANOVA followed by Tukey’s multiple comparisons test. **p < 0.01, ***p < 0.001, ****p < 0.0001.

To evaluate the extent of the MCP-1 gradient between culprit site and peripheral site, the fold change between both sites was calculated. An increasing MCP-1 ratio was indicative of relative fibrocyte accumulation at the site of occlusion (Figure 4A, *r*_*s*_ = 0.361, *p* = 0.013). The MCP-1 receptor CCR2 was proportionally less detectable on culprit site fibrocytes as shown by a negative correlation with the MCP-1 gradient (Figure 4B, *r*_*s*_ = −0.443, *p* = 0.030). However, the relative increase of culprit site dsDNA and citH3 was not associated with fibrocyte counts or their CCR2 expression (Supplementary Figure S5). Peripheral fibrocytes expressed significantly more CCR2 than culprit site fibrocytes (Figure 4C). Peripheral intensity of CCR2 expression remained stable over 72 h and was overall elevated compared to healthy controls (Figure 4C). CCR2 receptor expression at the culprit site was at median levels of about 60% of peripheral control (Figure 5A). PBMCs incubated with culprit site concentrations of MCP-1 *in vitro* showed minimal downregulation of the receptor CCR2 and only an increase of the dose far above physiological levels matched the extent observed *in vivo* (Figure 5B). To investigate a potential role of NETs in CCR2 receptor downregulation on fibrocytes, PBMCs were incubated with NETs and analyzed by flow cytometry. Culprit site levels of NETs significantly decreased CCR2 on fibrocytes compared to control supernatant to a degree observed for high MCP-1 dosages (Figure 5C). Addition of MCP-1 and NETs was even more potent than NETs alone (Figure 5C).

### *In vitro* Fibrocyte Migration Is Dependent on an MCP-1 Gradient

**FIGURE 6:**
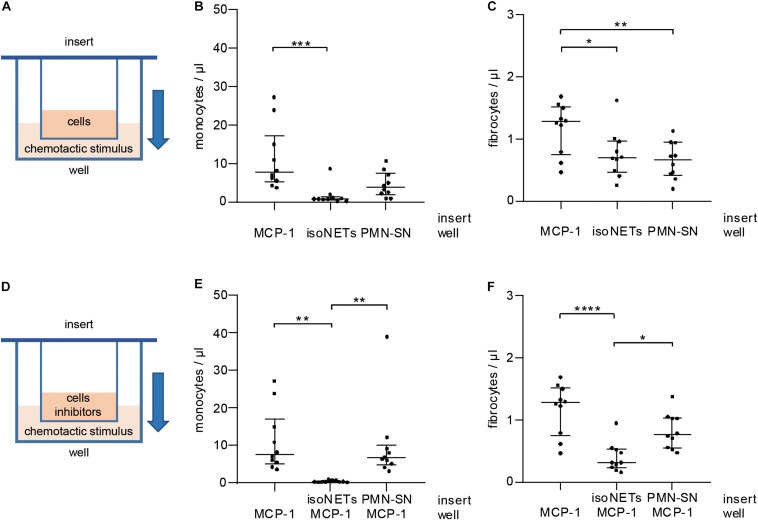
Influence of MCP-1 and NETs on monocyte and fibrocyte migration *in vitro.* **(A)** PBMCs were subjected to chemotaxis assays using cell culture inserts. MCP-1 (500 pg/ml), NETs (500 ng/ml dsDNA content), and neutrophil control supernatant (PMN-SN) were compared as chemoattractants (well). Chemotaxis was assessed by flow cytometry recording of **(B)** monocyte and **(C)** fibrocyte cell counts/μl. **(D)** NETs (500 ng/ml dsDNA content) and neutrophil control supernatant were investigated as inhibitors of MCP-1-mediated migration. Migrated cells were stained and counted by flow cytometry recording **(E)** monocytes and **(F)** fibrocytes/μl. Experiments were repeated with PBMCs and NETs of 10 healthy donors. Monocyte counts of graphs **(B,E)** were compared with Friedman test followed by Dunn’s multiple comparisons test, and fibrocyte counts of graphs **(C,D)** were analyzed by repeated measures ANOVA followed by Tukey’s multiple comparisons test. *p < 0.05, **p < 0.01, ***p < 0.001, ****p < 0.0001.

As NETs interfered with CCR2 receptor expression, we tested their influence on cell migration *in vitro* using cell culture inserts separating cells from the chemotactic stimulus by a porous membrane (Figure 6A). NETs did not act as a chemotactic stimulus for monocytes (Figure 6B) and were significantly less potent chemoattractants for fibrocytes (Figure 6C) than MCP-1. Control supernatant of unstimulated neutrophils did not exhibit significantly enhanced chemotactic properties when compared to NETs. Next, NETs were tested as inhibitors of MCP-1-mediated chemotaxis (Figure 6D). While monocytes could still migrate in response to MCP-1 when incubated with neutrophil control supernatant, presence of NETs in the cell suspension abolished any directed chemotaxis (Figure 6E). Fibrocyte migration was significantly reduced by NETs, but not by neutrophil control supernatant (Figure 6F).

## Discussion

**FIGURE 7:**
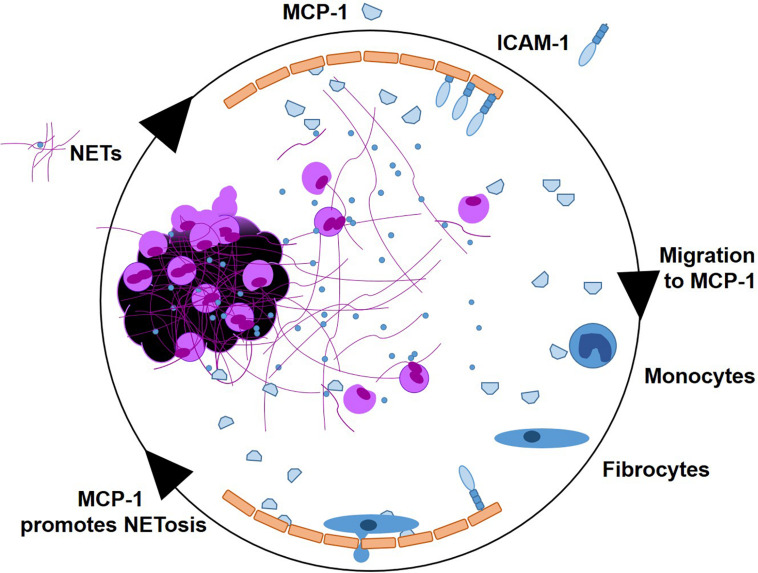
Graphical abstract. NETs as signaling scaffolds at the culprit site of STEMI. MCP-1 and ICAM-1 are expressed by endothelial cells. MCP-1 stimulates further NETosis. Monocytes enter the culprit site along an MCP-1 gradient, to transdifferentiate into fibrocytes in the presence of NETs.

In this study, we investigated pro-inflammatory effects of NETs at the culprit site of STEMI patients. We observed a marked local increase of the chemoattractant MCP-1 and the NET surrogate markers dsDNA and citH3 at the culprit site. MCP-1 promoted NET formation *in vitro* and NETs prompted a pro-inflammatory phenotype in hCAECs, inducing MCP-1 transcription and release as well as elevation of ICAM-1 and IL-6 mRNA expression. MCP-1 and NETs downregulated CCR2 receptor expression on fibrocytes *in vitro* and *in vivo.* NETs did not act as a chemotactic stimulus *in vitro* and seem to even suppress MCP-1-mediated migration. A graphical abstract is provided as Figure 7.

NETs dominate the acute inflammatory setting of STEMI (Mangold et al., 2015; Helseth et al., 2019). We reported that besides their cytotoxic (Saffarzadeh et al., 2012) and pro-thrombotic (Fuchs et al., 2012) properties, the presence of NETs affects other cell types such as fibrocytes (Hofbauer et al., 2019) and monocytes (Mangold et al., 2019) that are recruited to the culprit site and the ischemic myocardium. An important chemotactic molecule orchestrating immune cell recruitment in STEMI is MCP-1 (Prabhu and Frangogiannis, 2016).

Marked elevations of systemic MCP-1 levels in STEMI (Benson et al., 2013), NSTEMI, and patients with unstable angina (Wang et al., 2007) compared to healthy controls have been reported. Adding to these observations, we found MCP-1 to be highly increased at the culprit site, exceeding peripheral levels in STEMI. The time course of inflammation is reflected by a decline of MCP-1 in serial blood draws 72 h after STEMI in our study, which were still significantly higher than in healthy controls. Moreover, we could show that culprit site MCP-1 levels were significantly associated with infarct size in a multiple linear regression model complementing previous data on MCP-1 levels and patient survival after STEMI (de Lemos et al., 2007).

We found MCP-1 levels to be positively correlated with the NET markers dsDNA and citH3 at the culprit site; therefore, it was of interest to investigate potential causal relationships *in vitro*. MCP-1 is released by a variety of cell types such as smooth muscle cells, monocytes, and endothelial cells (Deshmane et al., 2009). As endothelium can be activated by neutrophil granule proteins (Soehnlein et al., 2009), we hypothesized that coronary artery endothelial cells would mount an inflammatory response in reaction to culprit site NETs. Indeed, we observed a potent manifold increase of MCP-1 mRNA and protein release by hCAECs when stimulated with culprit site levels of NETs. This effect seemed to be primarily mediated by neutrophil granule proteins attached to NETs, as we only observed an inferior impact of dsDNA and no effect by citH3. Moreover, treatment with human recombinant DNase 1 did not block MCP-1 release. In high concentrations, histones are cytotoxic for endothelial cells (Saffarzadeh et al., 2012). However, it is possible that signaling may require DNA and histones together as recently shown in monocytes (Tsourouktsoglou et al., 2020).

Our data suggest that MCP-1 is a component of the inflammatory cycle by enhancing and priming NET formation. As indicated by our *in vitro* data, MCP-1 promoted citrullination of histones and NET release upon a second stimulus. However, we were not able to link these observations to enhanced intracellular Ca^2+^ mobilization or ROS production in healthy donor neutrophils.

A mutual induction of NETs and MCP-1 is bound to attract other immune cells to the culprit site. Apart from previous reports on infarct-triggered infiltration of monocytes and macrophages (Dewald et al., 2005), we have recently shown accumulation of fibrocytes at the culprit site of STEMI with implications for ventricular dysfunction (Hofbauer et al., 2019). In this study, we report that relative fibrocyte influx seems dependent on the MCP-1 gradient between peripheral and culprit site. As MCP-1 is recognized by the chemokine receptor CCR2 (Luster, 1998), we measured CCR2 on fibrocytes to investigate fibrocyte trafficking in STEMI. CCR2 receptor expression was decreased on culprit site fibrocytes compared to the peripheral control. Furthermore, excessive increases of MCP-1 at the culprit site were associated with decreased CCR2 expression levels. To enable effective chemotaxis, leukocytes adapt receptor expression levels by uncoupling or internalization of receptors in response to ligand binding (Franci et al., 1996). Therefore, we hypothesized that fibrocyte CCR2 was downregulated at the culprit site in response to high MCP-1 concentrations, a process already described in monocytes (Handel et al., 2008;Volpe et al., 2012). Indeed, fibrocytes dose-dependently downregulated CCR2 *in vitro* when stimulated with MCP-1, however, the extent observed *in vivo* could not be achieved with physiological doses. Accordingly, we also investigated the effect of NETs on CCR2 expression. Incubation with culprit site concentrations of NETs led to a marked decrease of CCR2 on fibrocytes. To investigate whether NETs would also influence monocyte or fibrocyte chemotaxis, we performed *in vitro* migration experiments. NETs did not exhibit significant chemotactic properties in comparison to MCP-1 for fibrocytes or their progenitors, monocytes. Migration toward MCP-1 was even repressed by NETs added to the cell suspension. Chemotaxis by CCR2 signaling was inhibited in monocytes and fibrocytes. This is especially interesting as macrophages were recently reported to digest and then engulf NETs via macropinocytosis as mechanism of clearing (Haider et al., 2020). Cardiac repair demands a timely transition from early inflammation to wound healing without disproportionally prolonged or excessively unbalanced immune responses (Prabhu and Frangogiannis, 2016). We speculate that blockade by NETs could limit MCP-1-mediated accumulation to a certain degree but at the same time ensures extravasation of inflammatory cells into inflamed tissue. In support of this theory, addition of isolated NETs to hCAECs *in vitro* led to increased transcription of the adhesion molecule ICAM-1, an effect described in response to CAP37, an anti-microbial neutrophil protein, on human umbilical vein endothelial cells (Lee et al., 2003). Expression of CAMs is expected to facilitate extravasation of cells for subsequent clearance of dead cells in infarcted tissue enabling tissue repair (Prabhu and Frangogiannis, 2016). Furthermore, we have previously reported that NETs also enhanced the adhesion marker CD11b on fibrocytes (Hofbauer et al., 2019). CD11b facilitates leukocyte adhesion to the endothelium (Diamond et al., 1990). Consequently, fibrocytes were found increased in infarcted sections compared to healthy myocardium (Hofbauer et al., 2019), raising the possibility that accumulated fibrocytes contribute to adverse remodeling and scar formation by production of extracellular matrix in cardiac tissue.

Unfortunately, *in vitro* studies of NETs and their effects on receptors and signaling cascades are subject to certain limitations. Proteomic analysis of NETs formed spontaneously compared with in response to ionomycin, phorbol myristate acetate, and LPS exhibited only 22% protein homology (Petretto et al., 2019). Attached granule proteins and chemokines and thus the inflammatory setting possibly differ between diseases (Bruschi et al., 2019). However, our protocol for NET preparation using phorbol myristate acetate was associated with proteins of the immune response, interleukin signaling, degranulation, and cytoskeleton organization (Petretto et al., 2019). Detection of NETs in the circulation typically relies on markers that are not specific for NETs. dsDNA, for example, is a general marker of cell death (Pisetsky, 2016). The exception is citH3, which is firmly established as a specific marker for NETs (Savchenko et al., 2014; Warnatsch et al., 2015; Laridan et al., 2017; Thalin et al., 2017, 2019).

Mutual induction of MCP-1 and NETs shapes the inflammatory milieu of the culprit site in STEMI. Both mediators contribute to the early phase of inflammation and synergistically transition to fibrocytes for vascular healing and scar formation.

## Data Availability Statement

The raw data supporting the conclusions of this article will be made available by the authors, without undue reservation.

## Ethics Statement

The studies involving human participants were reviewed and approved by Ethics Committee of the Medical University of Vienna, Austria. The patients/participants provided their written informed consent to participate in this study.

## Author Contributions

TH, AO, and IL: conceptualization. TH, AO, AM, and CB: methodology. TH and AO: validation and TS formal analysis, writing—original draft preparation, and visualization. TH, AO, AM, TS, VS, and JN: investigation. AM, CB, and IL: resources. TH, AO, and VS: data curation. AM, TS, JN, VS, CB, and IL: writing—review and editing. AM and IL: supervision. IL: funding acquisition. All authors contributed to the article and approved the submitted version.

## Conflict of Interest

The authors declare that the research was conducted in the absence of any commercial or financial relationships that could be construed as a potential conflict of interest.

## Abbreviations

ACTB: actin beta
AUC: area under the curve
BSA: bovine serum albumin
CCR2: C-C chemokine receptor type 2
CD: cluster of differentiation
Cq: cycle of quantification
citH3: citrullinated histone H3
CK-MB: creatinine phosphokinase isoform MB (CK-MB)
DNase: Deoxyribonuclease
ELISA: enzyme-linked immunosorbent assay
FC: flow cytometry
FCS: fetal calf serum
FSC: forward scatter
hCAECs: human coronary artery endothelial cells
ICAM-1: intercellular adhesion molecule-1
IL-6: Interleukin-6
IQR: interquartile range
MCP-1: monocyte chemoattractant protein-1
MFI: mean fluorescence intensity
NETs: neutrophil extracellular traps
PBMCs: peripheral blood mononuclear cells
PBS: phosphate-buffered saline
ROS: reactive oxygen species
RT-qPCR: reverse transcriptase quantitative polymerase chain reaction
SSC: side scatter
SD: standard deviation
STEMI: ST-segment elevation myocardial infarction.
