# Supplementary material for: Neutrophil Extracellular Traps Induce MCP-1 at the Culprit Site in ST-Segment Elevation Myocardial Infarction
Source: Front Cell Dev Biol. 2020 Nov 9;8:564169. doi: 10.3389/fcell.2020.564169 (PMC7680894; doi:10.3389/fcell.2020.564169)
Supplement: Supplementary file 1 [file Data_Sheet_1.docx]

Supplementary Material

# Supplementary Figures


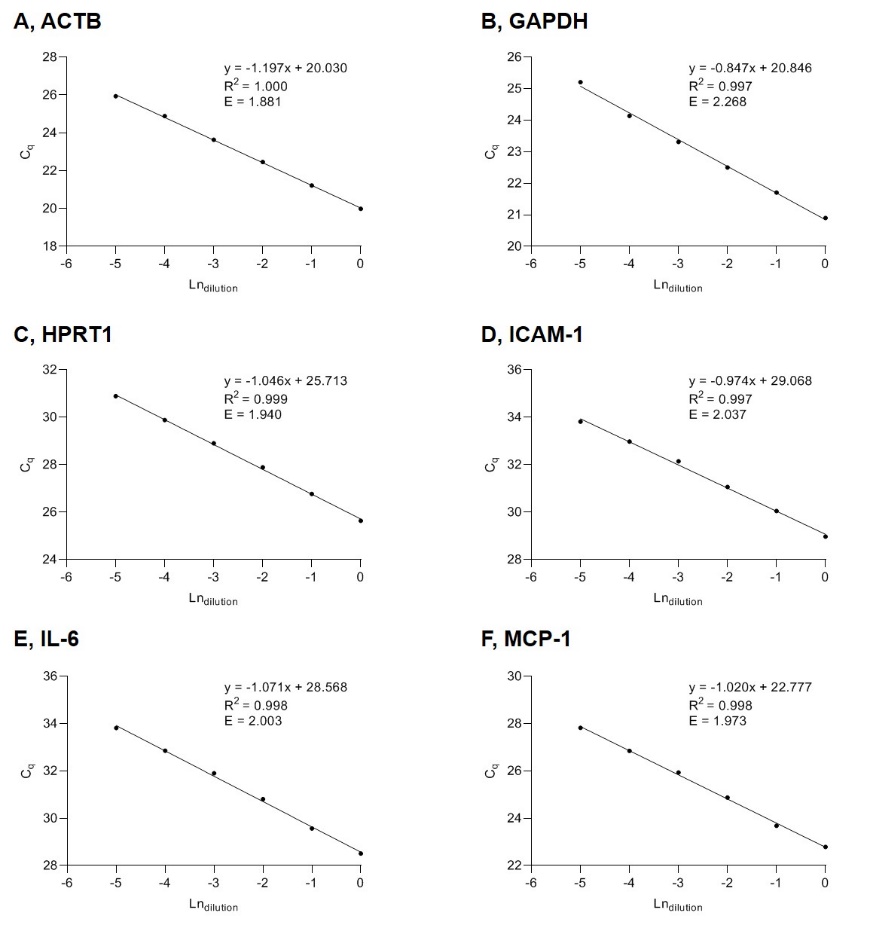


**Supplementary Figure S1 Efficiencies of used primer-probe-systems.**

Primer-probe-systems were established using serial two-fold dilutions of pooled cDNA to generate efficiency curves for Pfaffl correction in qPCR analysis (one experiment each).


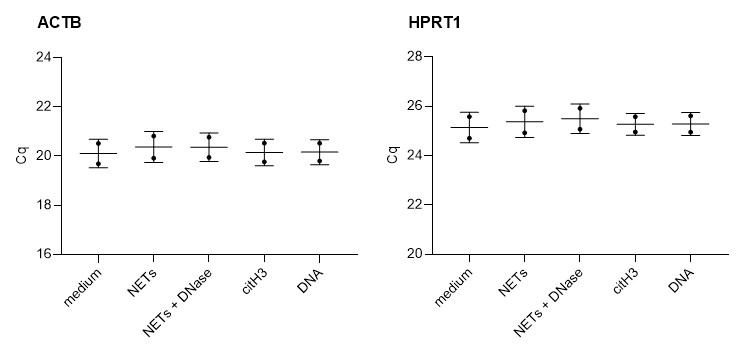


Supplementary Figure S2 Constant expression of reference genes independent of stimulation

mRNA of differentially stimulated hCAECs was extracted, transcribed into cDNA, and analyzed for stable expression of the reference genes ACTB and HPRT1 in qPCR to validate their use for normalization. Experiments were repeated two times.


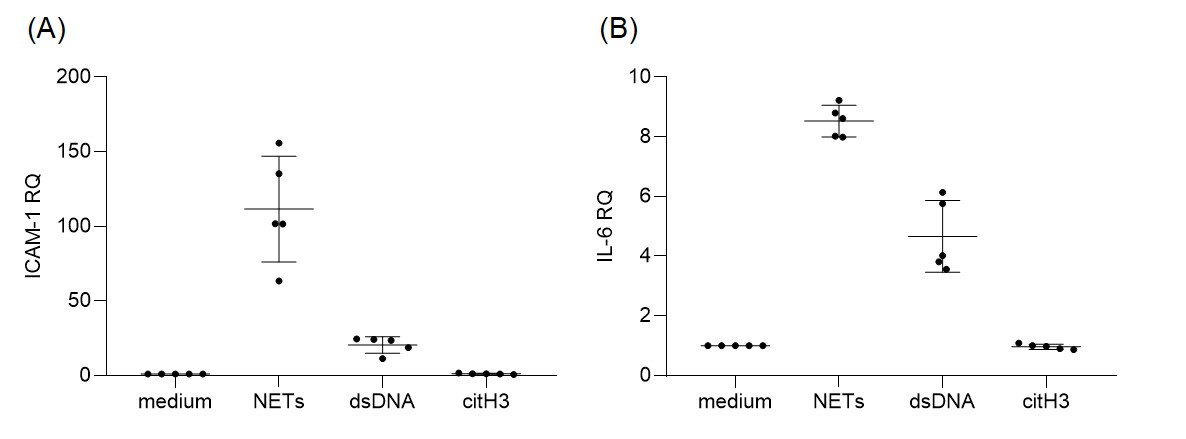
**Supplementary Figure S3 Stimulation of human coronary artery endothelial cells with NETs**

hCAECs were stimulated with isolated NETs, dsDNA, and citH3 for 6 and 24 hours. mRNA expression was assessed by qPCR after 6 hours for (A) ICAM-1, and (B) IL-6, and is presented as fold-change to an unstimulated control after normalization to the mean of two endogenous controls. Experiments were repeated 5 times.


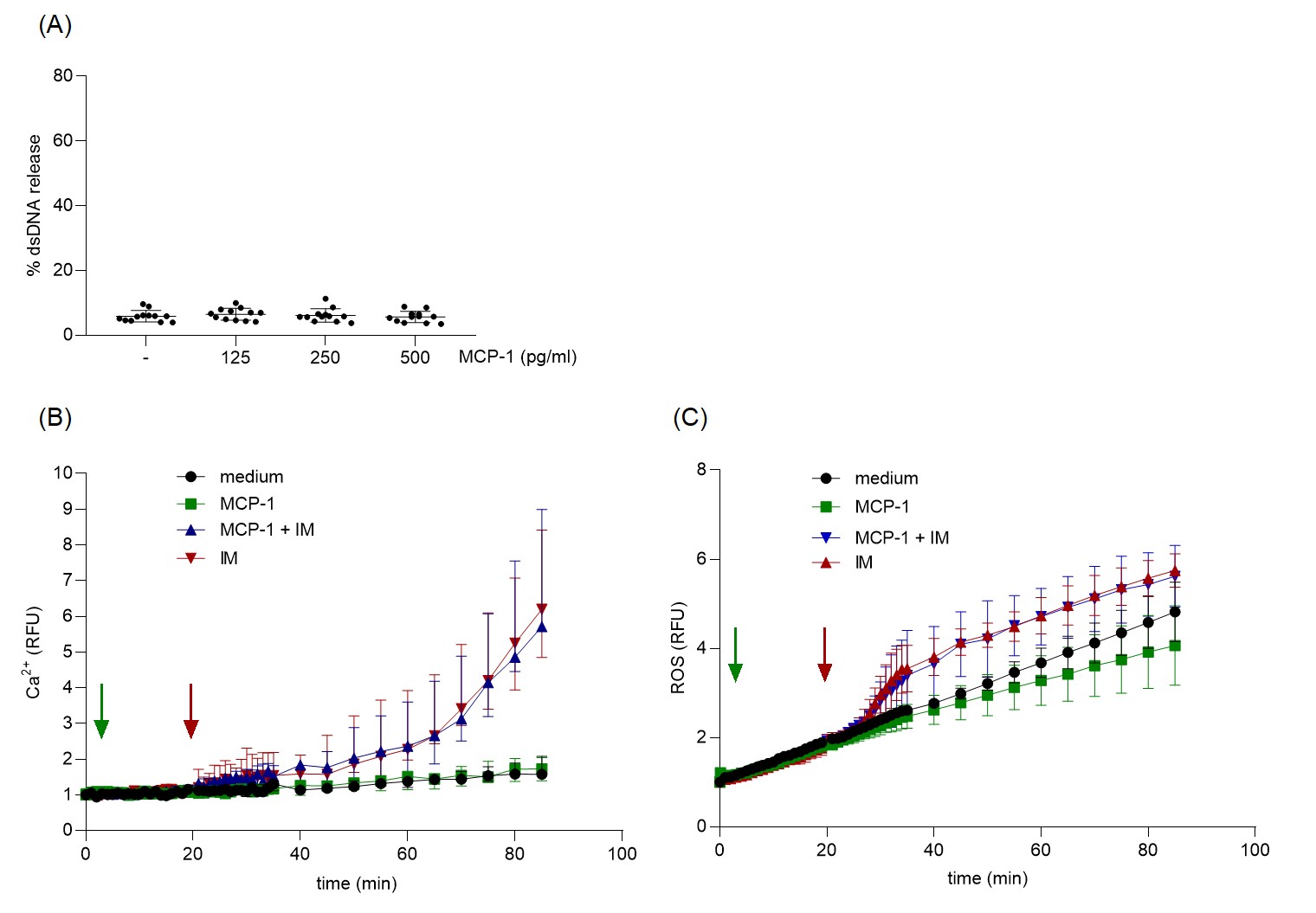


**Supplementary Figure S4 Effect of MCP-1 on NET formation, Ca^2+^ flux, and reactive oxygen species *in vitro***

(A) Neutrophils of healthy donors were treated with 125, 250, and 500 pg/ml MCP-1 to induce formation of NETs as indicated by release of dsDNA. Data are given in percent of positive control and are presented as mean ± SD. Experiments were repeated with 12 healthy donors and compared using the Friedman test (p=0.0321) followed by Dunn’s multiple comparisons test which did not indicate significant differences between specific groups. (B) Intracellular calcium flux of healthy donor neutrophils (n=7) was monitored after stimulation with 500 pg/ml MCP-1 (green arrow) and 1.3 µM ionomycin (IM, red arrow) for 85 min. (C) Intracellular formation of reactive oxygen species (ROS) in healthy donor neutrophils (n=2) was monitored after stimulation with 500 pg/ml MCP-1 (green arrow) and 1.3 µM ionomycin (IM, red arrow) for 85 min.


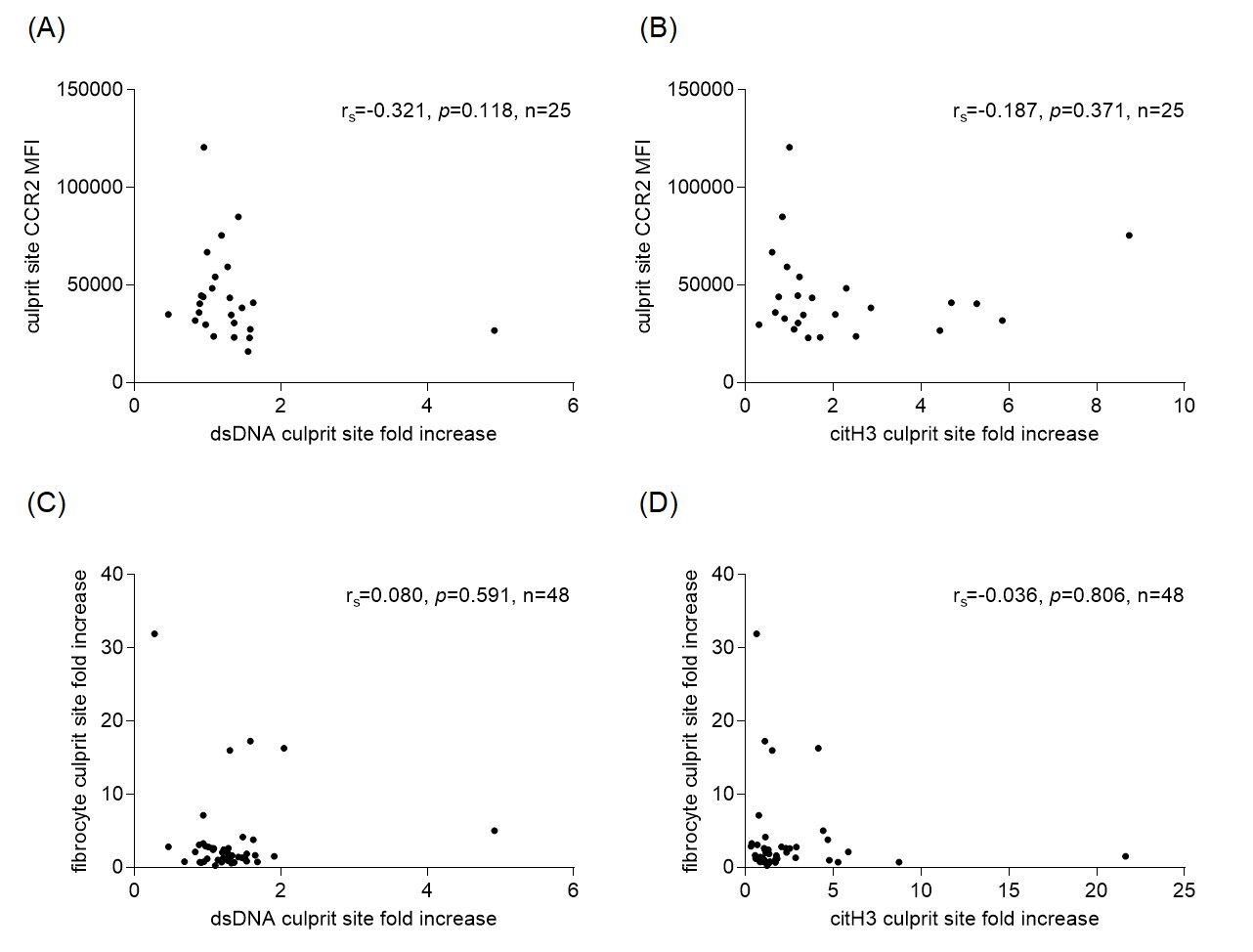


Supplementary Figure S5 dsDNA and citH3 fold increases are not associated with fibrocyte counts or their CCR2 expression

Fibrocyte counts and mean fluorescence intensity (MFI) of CCR2 expression was determined by flow cytometry. dsDNA and citH3 were measured in plasma and are presented as fold of peripheral levels at the culprit site. (A) Negative correlation of fibrocyte CCR2 expression at the culprit site with dsDNA fold increase (Spearman signed-rank test, r_s_=-0.321, n=25). (B) Negative correlation of fibrocyte CCR2 expression at the culprit site with citH3 fold-increase (Spearman signed-rank test, r_s_=-0.187, n=25). (C) Correlation of relative fibrocyte influx at the culprit site with MCP-1 fold increase (Spearman signed-rank test, r_s_=0.080, n=48). (D) Correlation of relative fibrocyte influx at the culprit site with citH3 fold increase (Spearman signed-rank test, r_s_=0.080, n=48).

# Supplementary Tables

Supplementary Table 1

| **FC target** | **Group 1** | **Group 2** | **Test** | **p-value** | **Corrected p-value** | **Indicator of significance** |
| --- | --- | --- | --- | --- | --- | --- |
| MCP-1 | peripheral site (n=47) | culprit site (n=47) | Wilcoxon matched-pairs signed rank test | 0.0001 | 0.0004 | *** |
| MCP-1 | peripheral site (n=47) | controls (n=15) | Mann-Whitney test | 0.0001 | 0.0004 | *** |
| MCP-1 | 72 h FU (n=19) | Controls (n=15) | Mann-Whitney test | 0.005 | 0.01 | * |
| MCP-1 | peripheral site (n=19) | 72 h FU (n=19) | Wilcoxon matched-pairs signed rank test | 0.0182 | 0.0182 | * |

Supplementary Table 2

| **FC target** | **Group 1** | **Group 2** | **Test** | **p-value** | **Corrected p-value** | **Indicator of significance** |
| --- | --- | --- | --- | --- | --- | --- |
| CCR2 | peripheral site (n=26) | culprit site (n=26) | Wilcoxon matched-pairs signed rank test | 0.0006 | 0.0024 | ** |
| CCR2 | peripheral site (n=26) | Controls (n=18) | Unpaired t- test with Welch's correction | 0.0049 | 0.0147 | * |
| CCR2 | 72 h FU (n=14) | Controls (n=18) | Unpaired t-test | 0.0111 | 0.0222 | * |
| CCR2 | peripheral site (n=14) | 72 h FU (n=14) | Paired t-test | 0.1766 | 0.1766 | n.s. |

Supplementary Table 3 List of materials and reagents

| **Product** | **Company** | **Catalogue number** |
| --- | --- | --- |
| Alu I | Roche | 14890823 |
| Anti.human Fc Block | BD Pharmingen | 564220 |
| Anti-human CCR2 | Biolegend | 357212 |
| Anti-human CD14 | Biolegend | 301838 |
| Anti-human CD34 | Biolegend | 343522 |
| Anti-human CD45 | Biolegend | 304014 |
| Anti-human CD66b | Biolegend | 305112 |
| Anti-human collagen-I | Merck-Millipore | FCMAB412F |
| Anti-human histone H3 (citrulline R2 + R8 + R17) | Abcam | ab5103 |
| BD FACS™ lysis solution | Becton Dickinson | 349202 |
| Bovine Serum Albumin Solution | Sigma-Aldrich | A7979-50ML |
| Cell Death Detection ELISA Plus kit | Roche | 11 774 425 001 |
| Citrullinated histone H3 | Cayman | 17926 |
| DCFDA Cellular ROS Detection Assay Kit | Abcam | ab113851 |
| K3EDTA-coated blood collection tubes | Greiner BioOne | 455036 |
| Fetal bovine serum | Merck | S0615 |
| Fluo-8 No Wash Calcium Assay Kit | Abcam | ab112129 |
| Goat anti-rabbit-Alexa Fluor 647 | Invitrogen | A21246 |
| Goat anti-rabbit HRP conjugate | BioRad | 170-6515 |
| GoScript Reverse Transcription System | Promega | A5000 |
| GoTaq® Probe 2-step RT-qPCR System | Promega | A6001 |
| HBSS without phenol red | Lonza | BE10-508F |
| HEPES buffer Solution (1M) | gibco | 15630-256 |
| Hetastarch 6% | B\|Braun | L6511 |
| Histopaque 1119 | Sigma-Aldrich | 11191 |
| Human coronary artery endothelial cells | Lonza | CC-2585 |
| Human MCP-1 DuoSet ELISA | R&D Systems | DY279 |
| Ionomycin | Sigma-Aldrich | I3909 |
| Lambda DNA | ThermoFisher | P7589 |
| L-Glutamine 200 mM | Lonza | BE17-605E |
| Lymphocyte Separation Medium | PromoCell | C-44010 |
| Medium 199 | Sigma-Aldrich | M4530 |
| MEM Non-essential Amino Acid Solution (100x) | Sigma-Aldrich | M7145-100mL |
| Pen/Strep, Amphotericin B (100x) | Lonza | 17-745E |
| Percoll | Sigma-Aldrich | P1644 |
| Phorbol myristate acetate | Sigma-Aldrich | P1585 |
| Phosphate-buffered saline | Sigma | D8537-500ML |
| Quant-iT PicoGreen kit | ThermoFisher | P7589 |
| Recombinant MCP-1 | Biolegend | 571404 |
| ReliaPrep RNA Miniprep System | Promega | Z6010 |
| RPMI-1640 Medium | Sigma | R8758-500ML |
| RPMI Medium 1640 without phenol red | Gibco | 11835-063 |
| Sulfuric acid | Sigma-Aldrich | 339741-100ML |
| Sytox Green | Invitrogen | S7020 |
| TMB Substrate solution | ThermoFisher | N301 |
| TRIzol™ reagent | ThermoFisher | 15596026 |
| Trypsin EDTA | Lonza | BE17-161E |
